# Implementation of a Sepsis Code Protocol at an Academic Institution in Colombia: A Pilot Study

**DOI:** 10.3390/jcm15020767

**Published:** 2026-01-17

**Authors:** German Devia Jaramillo, Laura María Castillo Morales, Camilo Antonio Vega Useche

**Affiliations:** 1 Emergency Department, Hospital Universitario Fundación Santafé de Bogotá, Bogotá 111321, Colombia; 2 School of Medicine, Universidad del Rosario, Bogotá 111321, Colombia; 3 Department of Critical Medicine and Intensive Care, Hospital Universitario Fundación Santafé de Bogotá, Bogotá 111321, Colombia; laura.castillo@fsfb.org.co (L.M.C.M.); investigacionmedcritica@fsfb.org.co (C.A.V.U.)

**Keywords:** sepsis, septic shock, emergency department, mortality

## Abstract

**Background/Objectives:** Sepsis is a critical medical emergency with significant morbidity and mortality, particularly in resource-limited countries. Effective strategies are essential to lower the high death rate. The sepsis code protocol recommends coordinated, structured, and prompt interventions for thorough patient care. This study aimed to compare in-hospital mortality rates after implementing the Sepsis Code protocol with those of a cohort of patients previously treated according to standard institutional guidelines. **Methods:** A pilot quasi-experimental study using a historical cohort design was conducted, involving patients with sepsis treated in an emergency department. Bivariate and multivariate analyses, as well as survival analysis, were conducted to evaluate the effectiveness of the intervention. **Results:** A total of 342 patients were analyzed. Among those who received the intervention, mortality was 13.4%, while in the control group, it was 22.5% (*p* = 0.042). Additionally, a protective association was found between the intervention and mortality (OR, 0.53; 95% CI, 0.29–0.94). Factors associated with increased mortality risk included lactate levels, SOFA score, septic shock presence, and history of diabetes. **Conclusions:** The implementation of the Sepsis Code in the emergency area showed an association with lower in-hospital mortality, especially in patients with septic shock. However, due to the study’s design, further research is needed to employ more robust methodologies and confirm the protocol’s applicability in the region.

## 1. Introduction

Sepsis is a medical emergency that causes high global mortality [1]. This mortality is especially significant in resource-limited countries [2]. In some publications with populations from low- to middle-income countries, mortality from sepsis is estimated at around 21.8%, and from septic shock between 45.6% and 56.4% [3,4]. Although there are increasingly more treatment tools, no treatment protocols have been shown to reduce sepsis mortality worldwide since 2001 [5] significantly. The problem is likely due in part to the lack of standardization in the diagnosis of sepsis [5] and especially to the heterogeneity in the presentation of patients, which necessitates individualized therapies [6].

While the future of sepsis treatment is geared towards individualized therapies, timely recognition remains the cornerstone of managing this disease and is therefore a priority in sepsis research [7]. Currently, multiple tools exist for timely recognition; scales such as qSOFA [8], NEWS [9], and IEWS [10] have been developed to identify early on in the population of patients with suspected sepsis those at higher risk of death, thus enabling the implementation of early therapeutic and diagnostic measures to improve clinical outcomes. On the other hand, the Surviving Sepsis Campaign project is designed to provide a series of recommendations for the recognition and treatment of sepsis that are regularly updated [11], whose implementation, according to the meta-analysis by Damiani E, et al. [12], has achieved a positive association in favor of the reduction in mortality (OR, 0.66; 95% CI, 0.61–0.72) [12], so it is necessary that health care centers know and apply these measures promptly.

To improve compliance with international recommendations, as well as to encourage diagnostic suspicion and timely recognition of patients with sepsis, since 2023 a hospital institution in Colombia has been developing a strategy for the recognition and care of patients with suspected sepsis that was initially applied to patients in the emergency department but that was later applied to the entire hospital institution called the sepsis code protocol.

This is why the objective of this work is to demonstrate the effects on clinical outcomes of implementing the sepsis code protocol and compare them with a historical cohort of patients before the protocol’s implementation, which serves as the control group. This was done during the first phase of implementing the protocol, specifically in the emergency department.

## 2. Methods

### 2.1. Studio Design

This is a quasi-experimental historical cohort pilot study to compare the effect on in-hospital mortality of implementing the sepsis code protocol (S) with a cohort of patients before its implementation.

For the creation of the control group (C), this study collected data from patients registered in the sepsis database of the Institute of Emergencies and Trauma (ISMET) of the University Hospital Fundación Santa Fe de Bogotá, Colombia, between 1 August and 30 December 2024. For the intervention group, data were collected from patients admitted to the sepsis code protocol from 1 April to 30 August 2025.

### 2.2. Eligibility Criteria

The study was conducted at a high-complexity university hospital in Bogotá, Colombia. Patients over 18 years of age admitted through the emergency department with a diagnosis of sepsis and/or septic shock, according to the diagnostic criteria established by the Sepsis-3 consensus [8], were included, regardless of their origin. Patients referred to other institutions, patients who received treatment at another healthcare facility before admission to the emergency department, patients without documented confirmation of infection, and pregnant patients were excluded from this pilot study. For the intervention group (S), all eligible patients were sequentially enrolled until the 4-month observation period was completed. For the control group (C), data from all eligible patients registered in the sepsis database (ISMET) during the 4-month control period were included.

All patients were sequentially enrolled until the minimum sample size for each group was reached. The 4 months immediately preceding the official start of the intervention protocol were excluded because, since January 2025, some education and dissemination campaigns related to the sepsis code had already been conducted among emergency personnel, and it was considered that this could introduce bias into the comparison of the groups.

### 2.3. Methodology

The diagnosis of infection was confirmed if the patient met at least one of the following criteria: (a) positive blood culture showing a non-colonizing or non-contaminating agent; (b) positive urine culture (more than 100,000 CFU/mL), accompanied by urinary symptoms or sepsis without another apparent cause; (c) positive culture of endobronchial aspirate, sputum, or bronchoalveolar lavage; (d) positive culture of ascitic fluid (>250 polymorphonuclear leukocytes per field); (e) positive stool culture; (f) or evidence of intra-abdominal collections or pulmonary consolidation in the absence of the above.

### 2.4. Definition of Sepsis

Sepsis was diagnosed according to the current definition of sepsis [8]. A case was classified as sepsis when infection was confirmed, and evidence of organ dysfunction was indicated by a score of 2 or more points on the Sequential Organ Failure Assessment (SOFA).

### 2.5. Definition of Septic Shock

Septic shock was defined according to the third definition of sepsis [8], it is a subcategory of sepsis in which circulatory and cellular metabolism disturbances are profound enough to increase mortality [8] significantly, and its diagnostic criteria are: hypotension, sustained requirement of vasopressors to maintain a mean arterial pressure (MAP) = 65 mmHg and a serum lactate level greater than 2 mmol/L [8].

### 2.6. Definition of Sepsis Code Protocol

This institutional strategy for early and coordinated response is activated upon suspicion of sepsis or septic shock. It operationalizes international recommendations through time-bound goal packages and continuous outcome monitoring.

The institutional sepsis code protocol emphasizes the early recognition of patients with suspected sepsis and additionally evaluates the probability of mortality to ensure appropriate prioritization and allocation of resources according to each person’s needs. The most important guideline in this protocol is the personalization of care, meaning that each patient receives what they need at the moment they need it. This approach contrasts with the previous institutional model, which focused solely on uniform care targets for all patients.

For the initial recognition phase, training sessions were provided to the nursing staff responsible for triage in the emergency department, with the aim of improving clinical suspicion of infection. Once the nurse identified a potential infectious process, a mortality prediction scale was applied immediately. Initially, the NEWS score [9] was used; however, the IEWS scale [10], recently validated in our country [13], is now also employed. If the patient was classified as high risk of death based on the score, they were entered into the sepsis code protocol and prioritized for evaluation by an emergency physician, whose role was to confirm the suspicion of infection and initiate interventions based on time sensitivity and the specific needs of each patient. In the first hour, it prioritizes serum lactate measurement, blood culture collection, and initiation of broad-spectrum antibiotics. During the first three hours of shock, the goal is to achieve hemodynamic stabilization through supportive care and vasopressors, aiming for a mean arterial pressure of ≥65 mmHg. The hemodynamic improvement strategies for patients in the intervention group were no different from those in the control group. Interventions were left to the discretion of the attending physician based on institutional protocols and international recommendations.

**Figure 1:**
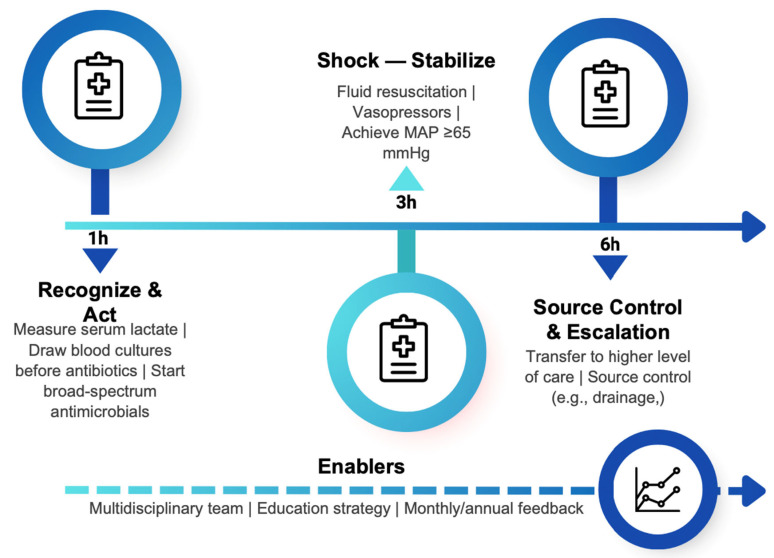
Sepsis code protocol: time-targeted actions (1-3 and 6 h) and enablers.

Within the first six hours, it includes transferring to a higher level of care and drainage of the infectious focus when appropriate. Its implementation relies on a multidisciplinary team and incorporates an educational strategy (education and dissemination campaigns) to raise awareness and train clinical staff, as well as a feedback system with monthly and annual outcome analysis to improve clinical care (Figure 1).

### 2.7. Sample Size

To calculate the sample size, a comparison of two proportions was used (mortality in the historical cohort vs. mortality in the intervention cohort). An approximate historical mortality of 23% was used [14,15], and it was expected to reduce mortality to 11%. Considering a two-tailed alpha of 0.05 and a power of 0.80, and initially assuming equal group sizes, a sample of 150 patients per group was obtained. Adding an estimated 10% loss, approximately 167 patients were identified per group. This calculation was performed using the R program, Version 2025.09.1+401, Copyright © 2025 by Posit Software, PBC, Version 2025.09.1.

### 2.8. Data Analysis

The data recorded in the data collection tool were reviewed to prevent inconsistencies or duplications, ensuring that the data matched each type of variable. A descriptive analysis of the study variables was conducted using categorical variables and frequency distributions. For continuous variables, measures of central tendency and dispersion were calculated based on the type of distribution (mean and standard deviation for normal distributions versus median and interquartile range (IQR) for non-normal distributions), with the Shapiro–Wilk normality test applied. Bivariate analyses focused on the mortality outcome. For categorical variables, the chi-square test or Fisher’s exact test was used to assess differences in mortality, depending on data distribution. For continuous variables, the t-test or Mann–Whitney U test was used to evaluate differences, depending on whether the data followed a normal distribution. Additionally, a multivariate logistic regression analysis was performed on the mortality outcome, including all variables from the database. The risk of hospital mortality was expressed as an odds ratio (OR) with a 95% confidence interval.

For the survival analysis, a Kaplan–Meier curve was created, and differences between the curves were assessed with the Log-Rank test. A *p*-value below 0.05 was deemed statistically significant. All analyses were conducted using R software, Version 2025.09.1+401, Copyright © 2025 by Posit Software, PBC.

## 3. Results

**Figure 2:**
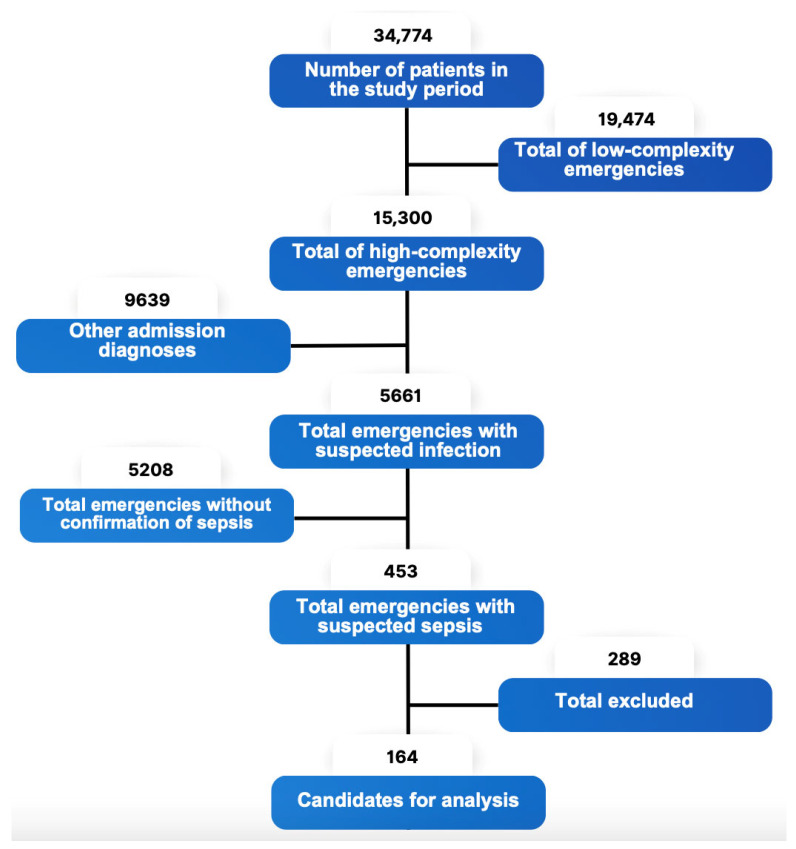
Flowchart for the inclusion of patients in the experimental protocol.

A total of 342 patients were analyzed, of which 164 patients with complete data were included in the analysis for the intervention group, while 178 patients were selected from the control group (Figure 2).

When comparing the study groups, no significant differences were found in age, sex, the number of patients with septic shock, or the number of patients with sepsis. No differences were found in lactate levels or SOFA scores between the intervention and control groups. Likewise, no differences were documented in the number of comorbidities between the patients in the compared groups. Finally, the most frequent site of origin of sepsis was pulmonary, followed by urinary, with no differences documented between the foci of infection and the compared groups (Table 1).

The overall mortality rate of the two cohorts was 18.1%. Multivariate analysis revealed that the variables associated with mortality were the presence of septic shock, lactate levels, a history of diabetes, and the SOFA score. Interestingly, the presence of sepsis without shock was associated with survival at the end of hospitalization, as was a history of immunosuppression. No association was found between mortality and the site of infection (Table 2).

**Figure 3:**
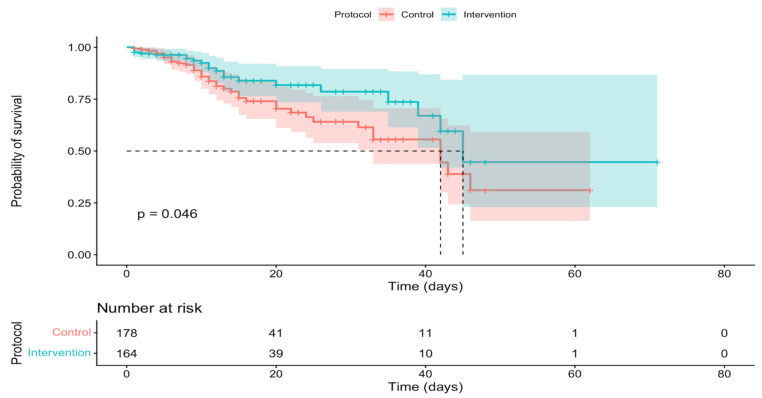
In-hospital survival curves according to protocol.

Finally, another variable linked to mortality was the type of protocol. The results of this study showed that the likelihood of in-hospital survival was significantly higher when the sepsis code protocol was used compared to the usual or control protocol (Figure 3).

Additionally, a statistically significant difference in mortality from septic shock was documented in the control group vs. the intervention group. Additionally, a higher mortality rate from sepsis without shock was observed in the control group compared to the intervention group, although this was not statistically significant (Table 3).

## 4. Discussion

In this quasi-experimental pilot study, the implementation of the sepsis code was associated with lower in-hospital mortality compared to the historical period (22.5% vs. 13.4%; OR 0.53; 95% CI: 0.29–0.94; *p* = 0.042), supporting the hypothesis that a structured protocol for early recognition and management could improve outcomes in sepsis and septic shock. The observed magnitude is clinically relevant, demonstrating a 9.1 percentage point decrease in mortality, equivalent to approximately 1 in 11 patients exposed to the Sepsis Code, potentially avoiding in-hospital death (exploratory estimate). In relative terms, this translates to a 40% reduction in risk (RR = 0.60) and a 47% reduction in odds (OR = 0.53). Our findings are consistent with reports where the implementation of the sepsis code was associated with lower mortality. In a Spanish ICU, the implementation of the program resulted in a reduction in in-hospital mortality from 44% to 23% and a decrease in 28-day mortality from 56% to 31% [16].

Similarly, in a study conducted in the Spanish Autonomous Community of Aragon, in-hospital mortality decreased from 31.1% to 20.7% and 30-day mortality from 30.1% to 19.8% after the implementation of the protocol [17]. On a large scale, adherence to the SEP-1 bundle (the Centers for Medicare & Medicaid Services early management bundle, which includes lactate testing, blood cultures before antibiotics, early antibiotics, 30 mL /kg of crystalloids if appropriate, vasopressors, and reassessment at 3–6 h windows) in patients reported to Medicare by 3241 hospitals in the United States was associated with lower 30-day mortality in a propensity score-matched study [18]. However, a recent systematic review found no moderate- or high-level evidence that SEP-1 adherence reduces mortality, and observational studies indicate that clinical complexity and management barriers may confound the relationship between adherence and mortality [19].

Taken together, the results of this study contribute to the growing body of evidence while acknowledging the variability due to adherence, case mix, and healthcare setting. The plausibility of the effect is consistent with the time-dependent nature of sepsis. The sepsis code reduces diagnostic and therapeutic delays, standardizing the administration of early antibiotics, fluids, vasopressors, and source control, as outlined in the Surviving Sepsis 2021 guidelines [11]. Delaying antibiotics increases mortality; in a New York City study, administering them between 3 and 12 h increased the odds of death by 14% compared to administration within 3 h [20]. Similarly, in septic shock, each hour of delay after hypotension was associated with worse survival [21]. This is why early vasopressor administration protocols may be justified, leading to a reduction in mortality [22,23].

In Latin America, published evidence on the implementation of structured sepsis care protocols, with formal impact assessment, remains limited and is mainly focused on Brazilian initiatives [24]. These gaps suggest that the present study is a pioneering contribution in the region by reporting an effect associated with the operational implementation of a sepsis protocol in an emergency department.

In the multivariate analysis, it was found that other variables significantly associated with mortality, besides not being in the intervention group, were elevated lactate levels, a data point already shared in multiple studies like this recent meta-analysis where lactate value was associated with mortality (Standardized mean difference pooled: 0.94; 95% CI: 0.34–1.54; I^2^ = 92%) [25], Other variables included the presence of septic shock, the SOFA score value, and this data is also consistent with published literature such as the meta-analysis by Lu et al. [26], where the Summary Receiver Operating Characteristic (SROC) was 0.819 (95% CI, 0.783–0.850), and finally, the last variable associated in the analyzed cohorts was having a history of diabetes, a data point that can also be compared with previous studies where glucose levels are associated with poor outcomes in septic patients [27]. When a patient experiences a severe infection such as sepsis, a state of stress occurs in which glucocorticoids, epinephrine, and even norepinephrine are released. This release leads to elevated glucose levels [28]. In individuals who cannot adequately manage high glucose levels, such as diabetic patients, these elevated levels can cause immunosuppression, inflammatory changes, endothelial cell dysfunction, nervous system injury, and oxidative stress [27]. This dysfunction could contribute to poor patient outcomes. On the other hand, diabetic patients may adapt to chronic hyperglycemia and, therefore, exhibit a relative intolerance to hypoglycemic episodes, these hypoglycemic episodes are associated with an increased risk of mortality [27].

This study, which is original in the country where it was conducted, provides evidence from an emergency service of an institution located in a region with medium to low resources, with a consistent application of the Sepsis-3 definition and a main finding that is consistent. However, the quasi-experimental design with a historical cohort is vulnerable to secular trends and residual confounding; therefore, the results should be interpreted as an association, rather than causation [29]. The results support the institutionalization of a sepsis code, accompanied by ongoing training, time audits (including antibiotics and fluids), and integration with antimicrobial stewardship programs, in line with the Surviving Sepsis 2021 guidelines and the Hospital Sepsis Program Core Elements (CDC). Given the global burden of 49 million cases and 11 million deaths in 2017, even modest process improvements could lead to significant population benefits. Finally, as authors, we recommend institutionalizing the Sepsis Code in multiple hospital settings, accompanied by continuous evaluation, process metrics, and studies that strengthen causal inference.

## 5. Limitations

Some limitations include the fact that this comparison involves two historical cohorts whose data were collected at different times. Nevertheless, the populations had similar characteristics, making them comparable and lending credibility to the results. Since there was no masking of the intervention, this study is considered quasi-experimental. It is also important to note that randomizing patients was not considered appropriate during the short-term pilot of the sepsis code protocol at the institution. Both groups were formed sequentially within the planned time frames, with no exclusions. Although the patient numbers between the groups may differ, the study met the initially planned sample size from the outset. During the assessment in the triage room, some patients were considered high risk using the NEWS scale and others using the IEWS scale, which could introduce some bias; however, it is worth noting that the severity of the disease in the two populations, as measured by the SOFA scale, was comparable.

## 6. Conclusions

This pilot study found that implementing the Sepsis Code in the emergency department was linked to lower in-hospital mortality, particularly among patients with septic shock. The multivariate analysis confirmed higher risks of death for patients with septic shock, elevated lactate levels, diabetes, and higher SOFA scores. However, given the study’s design, these findings should be seen as associations rather than proof of causation. To verify these results and assess their applicability across the region, more comprehensive multicenter research, such as cluster randomized trials or interrupted time series studies, is necessary.

## Figures and Tables

**Table 1:** Patient characteristics.

Variable	Control (%)	Intervention (%)	*p*
Total (n = 342)	178 (52)	164 (48)	
Sex (male, 58.8%)	107 (61.1)	94 (57.3)	0.678
Age (median(IQ))	72 (58–80)	72.5 (60–82)	0.636
Septic shock (40.9%)	66 (37.1)	74 (45.1)	0.161
Sepsis (59.1%)	112 (62.9)	90 (54.9)	0.161
Lactate levels (median (IQ))	2.4 (2–2.9)	2.3 (2–2.9)	0.498
SOFA (median (IQ))	3 (2–4.7)	3 (2–6)	0.197
**Comorbidities (%)**			
Cardiovascular (56.4)	91 (51.1)	102 (62.2)	0.051
Diabetes (21.6)	41 (23)	33 (20.1)	0.602
Renal insufficiency (9.9)	12 (6.7)	22 (13.4)	0.060
Immunosuppression (21.3)	32 (18)	41 (25)	0.147
Lung diseases (22.5)	37 (20.8)	40 (24.4)	0.504
Others (11.7)	18 (10.1)	22 (13.4)	0.435
**Sepsis origin (%)**			0.107
Pulmonary (29.5)	49 (27.5)	52 (31.7)	
Urinary (25.1)	50 (28.1)	36 (22)	
Abdominal (23.7)	36 (20.2)	45 (27.4)	
No documented focus (9.1)	23 (12.9)	8 (4.9)	
Soft tissues (5.8)	11 (6.2)	9 (5.5)	
Bacteremia (5)	7 (3.9)	10 (6.1)	
Gynecological (1.2)	1 (0.6)	3 (1.8)	
Osteomyelitis (0.6)	1 (0.6)	1 (0.6)	
Hospital stay (median (IQ))	9.5 (6–16)	10 (6–17)	0.851

**Table 2:** Mortality analysis of cohorts.

Variable	Survivors (%)	No Survivors (%)	OR	*p*
Total (n = 342)	280 (81.9)	62 (18.1)		
Sex (male)	161 (57.5)	40 (64.5)	1.33 (0.76–2.40)	0.383
Age (median (IQ))	72 (58–82)	73 (63.2–84.5)		0.444
Septic shock	95 (33.9)	45 (72.6)	5.10 (2.81–9.65)	<0.001
Sepsis	185 (66.1)	17 (27.4)	0.19 (0.10–0.35)	<0.001
Lactate levels (median (IQ))	2.3 (2–2.7)	3 (2.3–5.5)		<0.001
SOFA (median (IQ))	3 (2–5)	4 (2–7)		0.007
Intervention Protocol	142 (50.7)	22 (35.5)	0.53 (0.29–0.94)	0.042
**Comorbidities**				
Cardiovascular	157 (56.1)	36 (58.1)	1.08 (0.61–1.90)	0.885
Diabetes	54 (19.3)	20 (32.3)	1.99 (1.06–3.64)	0.038
Renal insufficiency	25 (8.9)	9 (14.5)	1.74 (0.72–3.85)	0.273
Immunosuppression	67 (23.9)	6 (9.7)	0.34 (0.12–0.79)	0.021
Lung diseases	64 (22.9)	13 (21)	0.90 (0.44–1.72)	0.877
Others	36 (12.9)	4 (6.5)	0.48 (0.13–1.27)	0.229
**Sepsis origin (%)**				0.167
Pulmonary	85 (30.4)	16 (25.8)		
Urinary	75 (26.8)	11 (17.7)		
Abdominal	68 (24.3)	13 (21)		
No documented focus	22 (7.9)	9 (14.5)		
Soft tissues	13 (4.6)	7 (11.3)		
Bacteremia	13 (4.6)	4 (6.5)		
Gynecological	3 (1.1)	1 (1.6)		
Osteomyelitis	1 (0.4)	1 (1.6)		

**Table 3:** Comparison of mortality by protocols.

Variable	Protocol		OR	*p*
	Control (%)	Intervention (%)		
Total in-hospital mortality	40 (22.5)	22 (13.4)	0.53 (0.29–0.94)	0.042
Mortality due to septic shock	27 (40.9)	18 (24.3)	0.46 (0.22–0.95)	0.035
Mortality due to sepsis	13 (11.6)	4 (4.4)	0.35 (0.11–1.12)	0.068

## Data Availability

The datasets used and/or analyzed during the current study are available from the corresponding author upon reasonable request.
